# Abnormal Uterine Bleeding in Perimenopausal Women: The Role of Hysteroscopy and Its Impact on Quality of Life and Sexuality

**DOI:** 10.3390/diagnostics12051176

**Published:** 2022-05-09

**Authors:** Salvatore Giovanni Vitale, Rafał Watrowski, Fabio Barra, Maurizio Nicola D’Alterio, Jose Carugno, Thozhukat Sathyapalan, Ilker Kahramanoglu, Enrique Reyes-Muñoz, Li-Te Lin, Bulent Urman, Simone Ferrero, Stefano Angioni

**Affiliations:** 1 Obstetrics and Gynecology Unit, Department of General Surgery and Medical Surgical Specialties, University of Catania, 95124 Catania, Italy; 2 Faculty of Medicine (Associate), University of Freiburg, 79106 Freiburg, Germany; 3 Academic Unit of Obstetrics and Gynecology, IRCCS Ospedale Policlinico San Martino, University of Genova, 16132 Genoa, Italy; fabio.barra@icloud.com (F.B.); simoneferrero@icloud.com (S.F.); 4 Division of Gynecology and Obstetrics, Department of Surgical Sciences, University of Cagliari, 09124 Cagliari, Italy; mauridalte84@gmail.com (M.N.D.); sangioni@yahoo.it (S.A.); 5 Obstetrics, Gynecology and Reproductive Sciences Department, University of Miami, Miami, FL 33146, USA; jac209@med.miami.edu; 6 Academic Diabetes, Endocrinology and Metabolism, Hull York Medical School, University of Hull, Kingston upon Hull HU6 7RX, UK; thozhukat.sathyapalan@hyms.ac.uk; 7 Department of Gynecologic Oncology, Emsey Hospital, 34912 Istanbul, Turkey; ilkerkahramanoglu@gmail.com; 8 Department of Gynecological and Perinatal Endocrinology, Instituto Nacional de Perinatología, Mexico City 11000, Mexico; dr.enriquereyes@gmail.com; 9 Department of Obstetrics and Gynecology, Kaohsiung Veterans General Hospital, No. 386, Dazhong 1st Rd., Zuoying Dist, Kaohsiung City 81362, Taiwan; litelin1982@gmail.com; 10 Department of Obstetrics and Gynecology, National Yang-Ming University School of Medicine, No. 155, Sec. 2, Li-Nong Street, Pei-Tou, Taipei 11265, Taiwan; 11 Department of Biological Science, National Sun Yat-sen University, 70 Lienhai Rd., Kaohsiung City 80424, Taiwan; 12 Centre for Reproductive Endocrinology and Infertility, American Hospital, 34365 Istanbul, Turkey; bulenturman@gmail.com; 13 Department of Obstetrics and Gynecology, Reproductive Endocrinology, Infertility Centre Istanbul, Koc University, 34450 Istanbul, Turkey

**Keywords:** abnormal uterine bleeding, hysteroscopy, perimenopause, quality of life, sexuality

## Abstract

Abnormal uterine bleeding (AUB) is a frequent symptom in perimenopausal women. It is defined as uterine bleeding in which the duration, frequency, or amount of bleeding is considered excessive and negatively affects the woman’s quality of life (QoL) and psychological well-being. In cases of structural uterine pathology, hysterectomy (usually performed via a minimally invasive approach) offers definitive symptom relief and is associated with long-lasting improvement of QoL and sexuality. However, over the past 30 years, uterus-preserving treatments have been introduced as alternatives to hysterectomy. Hysteroscopic polypectomy, myomectomy, or endometrial resection/endometrial ablation are minimally invasive techniques that can be used as an alternative to hysterectomy to treat AUB due to benign conditions. Although associated with high patient satisfaction and short-term improvement in their QoL, hysteroscopic treatments do not eliminate the risk of AUB recurrence or the need for further intervention. Therefore, considering the impact of different treatment options on QoL and sexuality during preoperative shared decision making could help identify the most appropriate and personalized treatment options for perimenopausal women suffering from AUB.

## 1. Abnormal Uterine Bleeding (AUB) in Perimenopausal Women

Perimenopause is the period between the first symptoms of diminished ovarian function, usually beginning in the early forties, lasting up to two years after the Final Menstrual Period (FMP). The variety and inconsistency of perimenopause definitions imply that a sharp distinction between “premenopausal”, “perimenopausal”, and “postmenopausal” AUB is difficult. In 1996, the World Health Organization defined perimenopause as “the period immediately prior to menopause (when the endocrinological, biological and clinical features of menopause begin) and the first year after menopause” [1]. In contrast, the term menopausal transition should be reserved for “that period of time before FMP when variability in the menstrual cycle is usually increased” [1]. Still, some studies arbitrarily set lower and upper limits of perimenopause, e.g., between 40 and 54 [2] or 42 and 52 [3] years of age or from four years before to 12 months after FMP [4].

**Figure 1:**
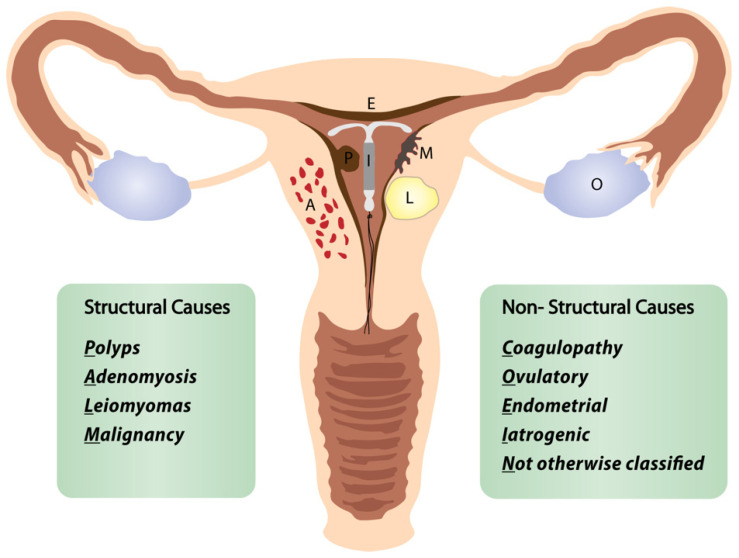
FIGO classification of abnormal uterine bleeding. Adapted from [8].

In this review, we define the “perimenopausal AUB” as any abnormal menstrual bleeding during menopausal transition or within the first year after menopause (except for cyclic bleeding in women using hormonal replacement therapy) [1,3,5]. AUB is the leading cause of approximately one-third of all outpatient gynecological visits, particularly in the perimenopausal period [3,4,5]. More than 90% of women experience at least one episode of AUB, and 78% of them at least three episodes of AUB during their transition to menopause [3]. The popular classification of nongestational causes of AUB was introduced by FIGO (International Federation of Gynecology and Obstetrics) in 2011 and revised in 2018 (Figure 1) [6,7,8]. The causes of nongestational AUB have been classified into nine categories arranged according to the acronym PALM-COEIN, including the structural causes (“PALM”): polyp, adenomyosis, leiomyoma, malignancy/hyperplasia, and non-structural causes (“COEIN”): coagulopathy, ovulatory dysfunction, endometrial, iatrogenic, and “not otherwise classified” [6,7]. The contribution of individual PALM-COEIN categories to the spectrum of AUB causes changes with age; nevertheless, endometrial polyps and fibroids remain the most common structural causes of AUB in perimenopause [9,10,11]. In the fourth decade of life, the impact of myomas and adenomyosis as AUB causes increases. Likewise, the highest incidence of endometrial polyps is reported in women aged 40–44 years [12]. Notably, the rate of structural pathologies (fibroids, polyps, adenomyosis) coexisting with each other or with uterine malignancies also increases with age [13,14]. Additionally, various local or systemic conditions, and hormonal or non-hormonal medications (tamoxifen, oral anticoagulants), can trigger uterine bleeding. Hematologic dysfunctions are also a frequent cause of AUB in perimenopausal women, being reported as the cardinal symptom in 32–100% of women with von Willebrand factor deficiency (relevant to 0.5–1% of the general population), in 5–98% of women with platelet dysfunction, and 35–70% of women with rare factor deficiencies [15]. Although the annual probability of spontaneous conception is about 10% by age 40–44, falling to 3% by age 45–49, the percentage of women fulfilling their reproductive goals in their fourth decade is continuously rising [16]. However, 84% of pregnancies in women over 48 years end in first-trimester miscarriage, and the rate of ectopics in women over 44 years rises to 7% [17]. Therefore, up to 12 months after FMP, excluding pregnancy is a mandatory part of the AUB diagnostic workup.

## 2. Initial Diagnostic Evaluation of AUB

The self-reported perception of AUB by the woman is the first step to determine its impact on QoL. It is well known that the patient’s subjective estimation does not always correlate with the objective amount of blood loss [18]. According to Munro et al. [6], menstrual bleeding exceeding 80 mL, as well as any intermenstrual and postcoital bleeding, should be considered abnormal. Nevertheless, about 14% of patients with mild to moderate blood loss consider their bleeding as heavy, and 40% of women with excessive blood loss consider their bleeding as standard [19]. In those cases, Pictorial Blood Assessment Charts can be helpful for the semiquantitative determination of AUB [20].

A detailed medical history (including hereditary disposition for uterine malignancies), the vaginal speculum exam, and transvaginal ultrasound (TVS) are essential parts of evaluating patients complaining of AUB [7,14,21]. However, standard diagnostic criteria and reproducibility of TVS are not consistent [14], and the accuracy of TVS in diagnosing benign uterine conditions is suboptimal [22,23]. TVS is helpful to exclude endometrial cancer (EC) in postmenopausal women if the endometrial echo is less than or equal to 4 mm, providing a negative predictive value of >99% [24]. The advantage of TVS is the holistic assessment of the uterus and its surrounding structures [7,24]. Saline infusion sonohysterography (SIS) offers a superior detection rate of benign lesions compared to TVS, but costs, convenience, and tolerability are the limiting factors [25,26]. Laboratory tests, e.g., hemoglobin and human chorionic gonadotropin determination, supplement the physical examination and sonography. Further laboratory tests may be indicated to uncover hereditary bleeding disorders or hormonal alterations depending on the history and the developed clinical suspicion.

## 3. Role of Hysteroscopy in the Management of Women with AUB

Hysteroscopy is considered the gold standard technique for diagnosing and managing pathological conditions affecting the uterine cavity [27,28]. In turn, AUB is the most common indication to perform hysteroscopy in perimenopausal women [29]. The hysteroscopic “see-and-treat” approach allows exploration of the uterine cavity, targeted endometrial and endocervical biopsies, and—if indicated—immediate treatment of endocervical, endometrial, or submucosal pathologies (polyps, myomas) [24,30,31,32,33,34]. It is essential to highlight that hysteroscopy is unsuitable for evaluating and treating deep myometrial pathologies (such as adenomyosis or myomas FIGO-Grade ≥ 3). Most hysteroscopic procedures can be performed in an office setting depending on the patient’s preferences, available infrastructure (staffing, equipment), surgeon’s experience, and comfort level [24,28,31,32,33,34]. More complex and prolonged procedures, such as hysteroscopic myomectomy and extensive lysis of intrauterine adhesions, are typically performed in the operating room, thus providing the patient with general anesthesia and the ability of the surgeon to perform more extensive surgery. Moreover, hysteroscopy can be complemented with laparoscopy if necessary [24,28,35,36]. Regardless of the setting in which the hysteroscopic procedure is completed, it is helpful to distinguish diagnostic and operative hysteroscopy.

### 3.1. Diagnostic Hysteroscopy

Diagnostic hysteroscopy aims to diagnose lesions within the endometrial cavity and, if necessary, to obtain targeted biopsies [24,28,37]. Diagnostic hysteroscopy can be a single intervention or may immediately precede hysteroscopic surgery. The feasibility of diagnostic hysteroscopy decreases in patients with previous surgeries, pelvic infections, IUD use, and postmenopausal status. Cobellis et al. [38] developed a predictive score for office hysteroscopy failure using different predictors, the most significant of which were history of procedures on the cervix, cesarean section, recurrent vaginitis, retroflexed uterus, and menopause. Depending on the previously indicated factors, office hysteroscopy could not be completed in 6 to 76% of the interventions [38].

**Figure 2:**
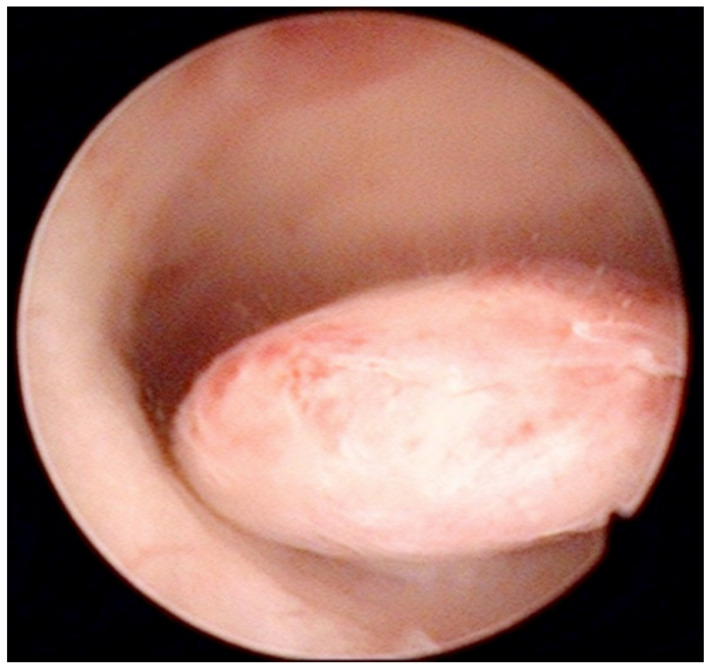
Pedunculate low-risk endometrial polyp in the posterior uterine wall during diagnostic hysteroscopy (intraoperative picture by F.B. and S.F.).

AUB is the presenting sign in >90% of postmenopausal women with EC [39]. In turn, the prevalence of EC or atypical hyperplasia in postmenopausal women with AUB is 21%, rising to 29% when AUB is accompanied by an endometrium thickness of ≥4 mm on TVS [39]. The systematic review by Clark et al. [40] confirmed high diagnostic accuracy of hysteroscopy with regard to EC, but only moderate for other types of endometrial disease. When comparing studies on the diagnostic accuracy of hysteroscopy, it is helpful to distinguish between studies reporting results based only on the hysteroscopic view and those obtained after a hysteroscopically-guided biopsy. For example, Elfayomy et al. [41] found “hysteroscopy” (meaning hysteroscopic image of the pathology) insufficient to exclude endometrial hyperplasia and cancer in women with AUB, based on 0.57/0.50 sensitivity and 0.92/0.94 specificity for endometrial hyperplasia and cancer, respectively (Figure 2). 

**Figure 3:**
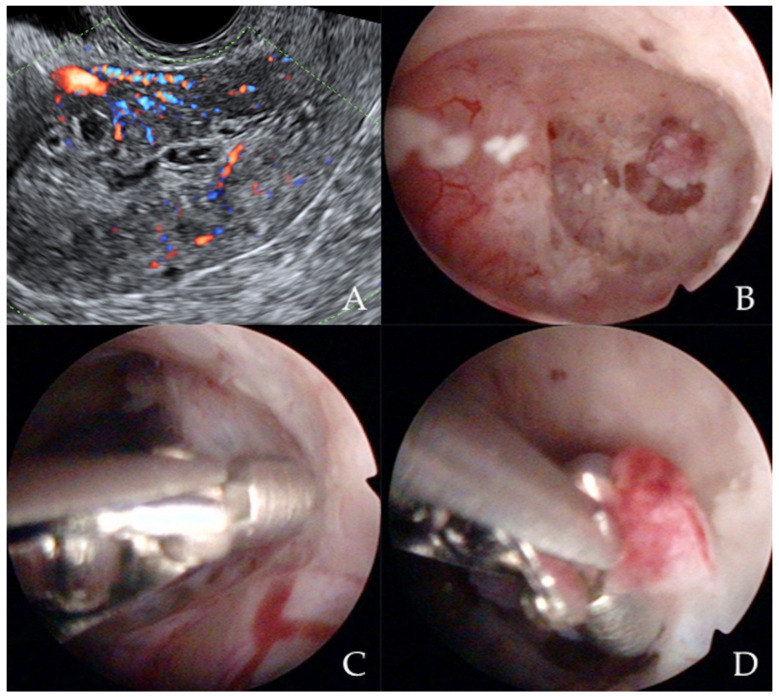
Ultrasonographic appearance of uterine lesion suspected for early-stage endometrial cancer (**A**). The diagnostic hysteroscopic allows for visualizing the suspected uterine area (**B**) and for obtaining the histologic biopsy (**C**,**D**). Intraoperative photographs by F.B. and S.F.

Similarly, Garuti et al. [42] used the terms “hysteroscopy” and “hysteroscopic view” interchangeably, reporting a low sensitivity (0.64 and 0.61) and specificity (0.92 and 0.95) for the diagnosis of endometrial hyperplasia. In the study of De Franciscis et al. [43], the concordance between the final histopathological result and the hysteroscopic impression was 86% for benign disease or normal endometrium, but only 58% for endometrial hyperplasia. In addition, the lowest agreement (52%) was noted for postmenopausal endometrial hyperplasia. When restricted to women with postmenopausal AUB, the sensitivity and specificity of in-office hysteroscopy for endometrial hyperplasia (with histopathology as reference) was 87% and 43%, respectively [43]. In contrast, Tinelli et al. [44] not only confirmed the diagnostic superiority of hysteroscopy with an eye-directed biopsy for detecting endometrial pathologies compared to TVS (and thus recommended hysteroscopy for all postmenopausal women with AUB and endometrial thickness > 4 mm) but also demonstrated the unique efficiency of hysteroscopy for diagnosing focal abnormalities (including EC) in the atrophic endometrium that would otherwise likely be missed by TVS. The authors advocate hysteroscopic evaluation even in patients with AUB with endometrium on TVS of <4 mm, aiming to decrease the chance of failing to diagnose carcinomas that develop focally in the atrophic endometrium (Figure 3) [44].

Less threatening but a more common finding in patients with AUB (20–31% of cases) are endometrial polyps [9,10,11,25,33]. The sensitivity of TVS for the diagnosis of polyps is particularly low (0.51) [25]. In contrast, in women with postmenopausal AUB, the sensitivity and specificity of hysteroscopy for the diagnosis of polyps are reported at 0.81–0.92 and 0.85–0.98, respectively [11,26]. The study by de Godoy Borges et al. [23] confirmed the superiority of diagnostic hysteroscopy (96.4% sensitivity, 74.6% specificity) in comparison to TVS (88.7% sensitivity, 25.4% specificity) for detecting endometrial polyps in women aged 41 to 82 presenting with AUB. Saline Infusion Sonohysterogram (SIS) provides a similar sensitivity compared with hysteroscopy but lower specificity (0.93 and 0.83 compared to 0.95 and 0.90, respectively). Finally, a recent meta-analysis reported sensitivity and specificity of 0.87 and 0.86, 0.62 and 0.73, and 0.92 and 0.85 for SIS, TVS, and hysteroscopy, respectively, for detecting endometrial polyps in women with AUB [11]. Similarly, hysteroscopy offers the highest diagnostic accuracy (>90%) for detecting submucous myomas as compared to TVS or SIS [45]. Further developments in the field of sonography, based on the three-dimensional virtual image synthesis (so called “virtual sonographic hysteroscopy”), will show to what extent diagnostic hysteroscopy could be replaced by next-generation, three-dimensional imaging modalities for detecting intracavitary uterine pathologies [46].

### 3.2. Operative Hysteroscopy

**Figure 4:**
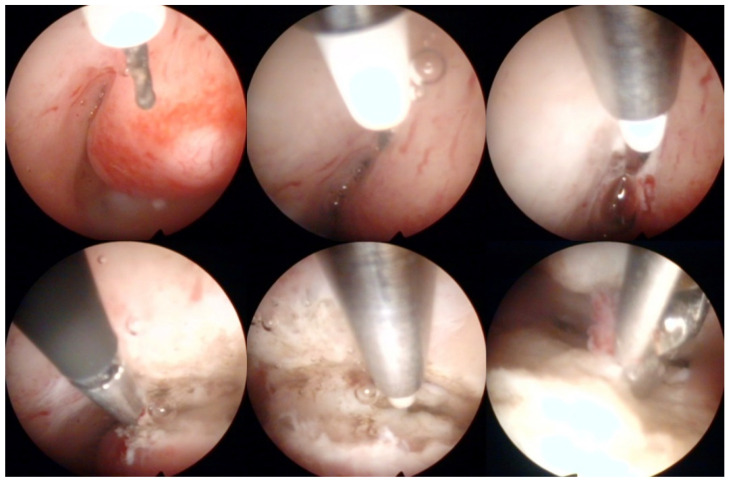
Operative hysteroscopic removal of an endometrial polyp (mean diameter 13 mm) by Versapoint system (Gynecare, Ethicon Inc., Raritan, NJ, USA). Intraoperative photographs by F.B. and S.F.

Operative hysteroscopy uses mechanical, electrosurgical, and laser instruments to treat intracavitary pathologies. The introduction of the small-diameter coaxial bipolar electrode (Versapoint, Gynecare, Ethicon, NJ, USA) in 1999 was a milestone for outpatient operative hysteroscopy (Figure 4) [47]. The development of miniaturized mechanical instruments with small diameter scopes and working channels with continuous flow systems enabled the “see-and-treat” approach without general anesthesia [32,34,48,49,50]. Wortman et al. [51] confirmed that major operative hysteroscopic surgery could be performed in an office-based setting resulting in a 98.8% rate of “satisfied” or “very satisfied” patients. Both outpatient (73%) and inpatient (80%) hysteroscopic polypectomy offer comparable success rates, as determined by the patients’ subjective bleeding and QoL assessment after six months [52]. However, procedure failure is higher (19% vs. 7%), and acceptability is lower (83% vs. 92%) with outpatient compared to inpatient polypectomy [52]. An essential aspect of hysteroscopic surgery is a very high level of physician satisfaction (e.g., 95% reported in [53]) associated with this approach. Factors such as incomplete resection and recurrence of the pathology decrease the acceptability of hysteroscopic surgery [38,52]. 

As with any other surgical procedure, hysteroscopic operations can be associated with complications. Every second complication of operative hysteroscopy is mechanical (52%), including cervical lacerations, uterine perforations, and injuries to the adjacent organs such as the bowel or bladder, sometimes associated with internal bleeding and conversion to laparoscopy or laparotomy [54,55]. Further short-term complications, such as excess fluid absorption, pulmonary edema, critical electrolyte disbalance, or genital tract burns, and long-term consequences (intrauterine adhesions) should be mentioned [54,55,56]. Venous air embolism during endometrial resection/endometrial ablation (ER/EA) or hysteroscopic myomectomy, although exceedingly rare (1:1140 surgeries), is a potentially fatal complication [57,58]. Myomectomies are hysteroscopic procedures with the highest complication rate (up to 14%) and the highest risk of distension medium-related complications (more than seven times more common compared to polypectomy) [54]. The application of monopolar energy is associated with more frequent local and systemic adverse events (perforations, burns, hyponatremia) as compared to bipolar energy [59,60]. Many of these complications can be prevented by strict adherence to basic surgical principles, e.g., limited use of monopolar devices [59,60]. Furthermore, some complications of intracavitary resections, e.g., intrauterine adhesions, can be successfully reduced by the use of antiadhesive barriers [61].

## 4. The Place of Hysteroscopy in the Treatment of the Perimenopausal Patient with AUB

The goal of AUB treatment in perimenopausal women—after the exclusion of an oncological cause—is to normalize menstrual blood loss and improve QoL [18,62]. The presence and severity of subsequent anemia influence the subjective experience of AUB and the therapeutic goals [62]. Irrespective of the underlying condition and planned surgical intervention, the treatment of anemia should be started as soon as it is recognized [18]. Clinical practice guidelines recommend early initiation of non-surgical methods such as tranexamic acid, oral or intramuscular progestins, gonadotropin-releasing hormone agonists, non-steroidal antiinflammatory drugs, or levonorgestrel-releasing intrauterine systems (LNG-IUS) [24]. From the QoL perspective, a patient’s preferences should be considered when selecting treatments. Before 1990, hysterectomy was considered the only available definitive treatment of AUB for women without a desire for future fertility [63]. Hysterectomy is associated with the resolution of the AUB symptoms and high satisfaction rates. Although minimally invasive approaches have significantly reduced perioperative morbidity, hysterectomy remains a major surgical procedure associated with severe complications [64,65]. Therefore, uterine sparing procedures should also be considered. Safe and cost-effective removal of myomas, polyps, and ER/EA for AUB treatment became possible via hysteroscopy and due to the evolution of mechanical or energy-based instruments [66,67,68]. However, uterine sparing procedures may require subsequent reinterventions [66,69].

## 5. Hysteroscopy for Perimenopausal AUB: Impact on QoL and Sexuality

When considering the mutual relation between hysteroscopy, AUB, QoL, and sexuality, it is necessary to address the interplay of the following factors: bleeding (with/without anemia), underlying conditions (e.g., menopausal status, cancer), treatments (effectiveness, discomfort caused during the procedure), over- or undertreatment (need to perform surgical procedures), age and menopausal status (regardless of AUB or its treatments), and the controversy about the impact of hysterectomy (regarding uterus-preserving techniques) on QoL and sexuality.

Furthermore, the impact of AUB on individual QoL and sexuality should be seen in the broader context of the menopausal transition, a phase marked by hormonal, physical, mental, sexual, biological, and social changes and an increasing prevalence of non-gynecological diseases. All of these changes can potentially impact women’s QoL and sexuality negatively. More than 80% of perimenopausal women experience complaints affecting QoL [70], and more than 60% of them suffer from three or more symptoms [71]. Additionally, of women aged 40–45 years, 32% experience heavy menstrual bleeding. These women have significantly worse QoL [72]. Perimenopausal symptoms critically impairing QoL include sleep disturbances, fatigue, and anxiety [71]. AUB can further compromise the overall health status and the health-related QoL (HRQOL), its sequelae (e.g., iron deficiency anemia, chronic fatigue), or associated comorbidities (e.g., pelvic pain) [62,72,73,74,75,76]. The treatment of endometriosis, myomas, and polyps leads to the best HRQoL scores, while treating nonspecific pelvic pain and AUB is less frequently associated with HRQoL improvement [75]. Post-AUB anemia has a detrimental impact on a woman’s QoL by interfering with physical activity, vitality, cognition, work performance, and social and emotional life [62,73,77]. In addition, age alone is one of the strongest independent factors compromising the QoL in women with AUB. 

Lastly, it is essential to highlight that HRQoL is substantially reduced in patients experiencing the combination of AUB and pain [73]. AUB associated with uterine fibroids is responsible for a significantly lower HRQoL than women without fibroids [78]. In premenopausal patients with menorrhagia, concomitant pain, mood changes/fatigue, and general malaise are the most bothersome concomitant symptoms in 27.4%, 17.4%, and 10.8% of patients, respectively [74]. 

As well as physical complaints, fear of cancer is also common among women with AUB. Timmermanns et al. [79] reported that 100% of postmenopausal women presenting with bleeding wanted to be sure that cancer is not the cause. Only 5% of women were willing to accept more than a 5% risk of cancer and not having intervention. Moreover, if the risk of recurrent bleeding due to benign disease exceeded 25%, most women would prefer immediate treatment of benign lesions instead of surveillance [79]. Since age is an independent risk factor for malignancy, several international boards (e.g., British, American, Canadian) recommend endometrial biopsies in women over 40 or 45 who have failed conservative treatments [18]. 

Once pregnancy, neoplasia, or hyperplasia are excluded, AUB treatment should always aim to improve the individual’s QoL [76,80]. For a reliable assessment of QoL, psychological well-being, or sexual function, the use of validated psychometric tools such as SF36 [73,80,81], WBQ-12 [82], or FSFI [70] is recommended. Vitale et al. [80] proposed a three-step multidisciplinary approach to AUB in perimenopausal women, considering at each stage the impact of the disease itself, the diagnostic approach, and specific treatment options. Table 1 provides an overview of this approach.

## 6. Procedure-Specific Factors Influencing QoL

Hysteroscopy is generally well accepted by patients; however, fear of the procedure and anticipated pain are the two most important factors. Sorrentino et al. [83] reported that 27% of women undergoing outpatient hysteroscopy had mild pain, 33% moderate pain, and 40% severe pain during the procedure. In 43% of those patients, analgesia was required. Preprocedural anxiety and perceived stress levels negatively affect the pain experienced during and soon after office hysteroscopy [83,84]. Among the non-modifiable factors, patient age and menopausal status positively correlate with hysteroscopy-related pain [85]. Of the modifiable factors, the length of waiting time before the procedure negatively influences pain perception [84,85]. The longer the time, the worse the pain reported by the patients during the procedure. Therefore, reducing waiting times, providing detailed information about the method, and reassuring the patient that the operation will be aborted at her request are effective measures to reduce anxiety and alleviate the pain during hysteroscopy [80,84,86]. The use of multimedia has improved patient comprehension, reduced fear about the procedure, and increased patient satisfaction. Before office hysteroscopy, a video-based multimedia informative session was the preferred method for lowering preprocedural anxiety and improving patients’ satisfaction [87].

The individualized approach to AUB should always prioritize patients’ preferences. ER/EA improves HRQoL and high satisfaction rates in women with AUB [88]. The complete resolution of AUB symptoms after hysteroscopic polypectomy ranges from 73 to 100% at follow-up intervals between 2 and 52 months [52,89]. Similarly, in women suffering from AUB due to intrauterine polyps or myomas, hysteroscopic removal using the MyoSure^®^ device in an outpatient setting provided significant and sustained HRQoL improvements up to 12 months after the procedure [90]. As reported by Laughlin-Tommaso et al. [91], hysteroscopic myomectomy enables fast return to daily activities, substantial improvements in short-term HRQoL, and lowering of the severity of the symptoms comparable to the outcomes of the laparoscopic or abdominal approaches. Nevertheless, the reliability of the cited analysis is questionable because the mean number and diameter of the myomas removed hysteroscopically were lower than those removed with laparoscopic or abdominal procedures. Following hysteroscopic myomectomy, the proportion of women with persistent symptoms dropped from 92% to 51%, and the overall burden was reduced by half [91].

### 6.1. AUB, Hysteroscopy, and Sexuality

The impact of AUB on sexual function during perimenopause is barely addressed in the literature. It is known that sexuality in perimenopause is influenced by changes in partnership, body image, general health, hormonal factors (estrogen and testosterone), increased prevalence of depressed mood, sleep disturbances, and vaginal dryness [92]. In the study of Trento et al. [92], 64% of women aged 40 to 65 years were at risk of sexual dysfunction, with lower scores in the domains of sexual desire and interest, comfort, orgasm, and satisfaction. Similarly, 70% of Polish perimenopausal women reported sexual dysfunction when assessed by FSFI [70]. However, impaired sexual function is less distressing for menopausal than for premenopausal women [93]. The additional contribution of AUB to sexual health is underreported. In women in late reproductive age suffering from AUB, the scores for sexual function reached only 55–69 out of 100 [94]. Marnach et al. [95] demonstrated that female sexual function improved and personal distress decreased after ER/EA for AUB. In contrast, Zhang et al. [96] compared the impact of laparoscopic and hysteroscopic myomectomy on sexual desire, sexual arousal, vaginal lubrication, orgasm, sexual satisfaction, and sexual intercourse pain in women at 3 and 6 months after the operation and did not observe any change before and after treatment independently of surgical modality or patients age. Notably, similar levels of sexual activity and sexual satisfaction were reported in women who underwent hysterectomy and hysteroscopic techniques [97,98,99].

### 6.2. Hysterectomy versus ER/EA

The role of QoL and the importance of an individualized approach to AUB is essential when offering uterine-preserving treatment for AUB versus hysterectomy. Hysterectomy is not the only treatment for AUB due to benign pathology. Therefore, the patient’s preferences and concerns, short- and long-term complications, and benefits of each treatment, including AUB recurrence, anemia, or progression from endometrial hyperplasia to carcinoma, and the implications for quality of life and sexuality, should be discussed during patient’s counseling and decision making. ER/EA is a minimally invasive alternative to hysterectomy for patients wishing to preserve their uterus [67]. However, according to the latest Cochrane Review, ER/EA results in lower resolution rates of symptoms, less patient satisfaction, and similar rates of serious adverse events compared to both open and minimally invasive hysterectomy. In addition, EA/ER results in poorer QoL and up to a 7.5-fold increased risk of further surgical intervention due to treatment failure compared to hysterectomy [66]. In an English cohort study of 114,910 women undergoing ER/EA, 16.7% had at least one subsequent procedure within five years [69]. Nonetheless, ER/EA shortens the return time to normal activities compared to open hysterectomy [66]. 

Regarding hysterectomy, postoperative complications (Clavien-Dindo grade ≥ III) and reoperations associated with hysterectomy are currently reported at 4% and 2.1%, respectively [65]. A complication usually related to sexual intercourse is vaginal cuff dehiscence, observed after 0.6–1.35% of TLHs and 1.6% of robotic hysterectomies [64]. The higher costs of hysterectomy and the longer duration of hospital stays should be outweighed against long-term costs related to AUB (anemia, fatigue, repeated treatments, etc.). Both hysteroscopic surgery and hysterectomy significantly reduce anxiety and depression without differences in women’s mental health at 12 months after surgery [97]. Additionally, psychological and social outcomes improve substantially one year after endometrial resection and hysterectomy without a significant difference between the treatment groups [100]. 

A recently published 10-year follow-up study confirmed that hysterectomy produces the most significant improvement in decreasing stress, discomfort and symptoms, and sexual activity, both in the short- and long-term [101]. Hysterectomy performed for benign conditions has beneficial effects on sexual function and overall well-being, regardless of the surgical technique [67,102,103,104], although 10–20% of women may experience deterioration in sexual function, such as dyspareunia or an altered orgasmic response [102]. In a survey performed 12 months after hysterectomy, over 96% of women did not regret having had the hysterectomy [105]. Radosa et al. [103] prospectively examined sexual function in patients undergoing vaginal, laparoscopic supracervical (LSH), and total laparoscopic hysterectomy (TLH). They found that all procedures led to an improvement in postoperative sexuality without significant differences between the procedures. Improvement in sexual functioning after LSH and TLH were observed by Berlit et al. [104]. The beneficial effects of hysterectomy on QoL and sexual health are likely attributed to the absence of vaginal bleeding, coital pain, and contraception-related issues [102].

### 6.3. Role of Hysteroscopy in Individualized Treatment of Uterine Malignancies

Lastly, the hysteroscopic treatments in cases of atypical endometrial hyperplasia [106], EC [107], or rare uterine tumors [108] have been reported. If uterus-sparing treatment of atypical endometrial hyperplasia or EC is considered, hysteroscopy constitutes both the primary surgical modality and, in combination with serial endometrial biopsies, the cornerstone of follow-up [109,110]. To date, uterus preservation in the presence of malignancy is controversial and cannot be recommended. However, the upcoming molecular classification of EC is expected to support the selection of patients eventually suitable for fertility-sparing treatments of early, low-grade malignancies [111].

## 7. Conclusions

AUB is a common symptom in perimenopausal women. Accurate diagnosis is essential to rule out pre-malignant or malignant conditions and provide the most appropriate treatment, taking into account the impact of both the disease and treatment on QoL and sexuality. Hysteroscopy offers a minimally invasive way to diagnose and treat intrauterine pathologies, and is well-tolerated, but it carries the potential risk of symptom recurrence and repeated surgeries. In contrast, hysterectomy enables definitive treatment of AUB and underlying conditions and long-standing improvement in QoL and sexuality but is more invasive than hysteroscopic treatments. In order to make the best treatment choice for the individual patients, short-term and long-term goals, including patient safety, QoL, and sexual well-being should always be considered.

## Figures and Tables

**Table 1:** Three-step multidisciplinary approach for postmenopausal women with intrauterine pathologies *.

Step	Key Points
1	Introduce yourself and get a problem-oriented, appropriate and relevant medical history
	Assess the impact of AUB on QoL using specific questionnaires, e.g., the 36-Item Short-Form Health Survey (SF-36) or Menorrhagia Multi-Attribute Scale (MMAS)
	Assess the implications of the gynecological symptoms, including sexuality, using e.g., Female Sexual Functioning Index (FSFI)
2	Manage the patient’s anxiety and stress related to the possible diagnosis
	Provide reliable disclosure of the malignant diagnosis
	Provide appropriate pain management during diagnostic/therapeutic surgical interventions
3	Identify the most appropriate therapeutic option according to the patient’s preferences
	Ensure adequate psychological support, especially for women with cancer diagnosis

* Table modified by Vitale SG et al. [80].

## Data Availability

Not applicable.
